# Case report: A rare case of catastrophic Takayasu arteritis: acute ischemic stroke and anterior ischemic optic neuropathy

**DOI:** 10.3389/fstro.2024.1481940

**Published:** 2024-11-28

**Authors:** Oladotun V. Olalusi, Joseph Yaria, Akintomiwa Makanjuola, Rufus Akinyemi, Mayowa Owolabi, Adesola Ogunniyi

**Affiliations:** ^1^Department of Neurology, University College Hospital, Ibadan, Nigeria; ^2^Neuroscience and Aging Research Unit, Institute of Advanced Medical Research and Training, College of Medicine, University of Ibadan, Ibadan, Nigeria; ^3^College of Medicine, University of Ibadan, Ibadan, Nigeria; ^4^Center for Genomics and Precision Medicine, College of Medicine, University of Ibadan, Ibadan, Nigeria; ^5^Department of Medicine, Lebanese American University of Beirut, Beirut, Lebanon; ^6^Department of Medicine, Blossom Specialist Medical Center, Ibadan, Nigeria

**Keywords:** Takayasu arteritis (TAK), vision loss, Stroke, acute ischemic stroke (AIS), anterior ischemic optic neuropathy (AION), large-vessel vasculitis, Nigeria, West Africa

## Abstract

Acute ischemic stroke (AIS) is a known complication of Takayasu arteritis (TAK); however, it is infrequently the first symptom observed. There have been no reports of concomitant AIS and anterior ischemic optic neuropathy (AION) as an initial manifestation of TAK. We present a case of TAK causing stroke and painless vision loss in a young Nigerian woman. A 33-year-old female patient presented with abrupt onset weakness in the right extremities and painless vision loss in her left eye. Her medical history included joint pain, malaise, syncope, and dizziness, along with peripheral vascular collapse, requiring recurrent hospital admissions. She had an absent left radial pulse, reduced left carotid pulse, and unrecordable blood pressure in her left arm. There was a relative afferent pupillary defect (RAPD), with fundoscopy findings indicating optic disc pallor. She had expressive aphasia, right facial paresis, and right flaccid hemiparesis. Brain computed tomography (CT) showed an infarct in the left middle cerebral artery (MCA) territory. The brain CT angiography showed diffuse enhancing aortic arch wall thickening and multiple aortic arch branch obstructive disease. The diagnosis was TAK complicated by left hemispheric infarctive stroke and left AION. She began treatment with prednisolone, azathioprine, and secondary stroke preventive care. Her vision improved to the ability to count fingers, with good functional outcomes and reduced disease activity. This case highlights the challenging diagnostic trajectory of TAK in a Nigerian female patient, featuring a unique multi-vessel affectation. Clinicians should be aware of the protean clinical presentations of TAK to reduce adverse cardiovascular complications.

## Introduction

The prevalence of stroke in young adults is increasing, with disproportionately large economic implications compared to stroke in older adults (Smajlović, 2015). Globally, valvular and congenital heart diseases, viral infections, substance abuse, and vasculitis are some of the known causes of stroke in young individuals (Smajlović, 2015). In sub-Saharan Africa, hemoglobinopathy and HIV are well-documented causes of stroke in young people, while in Asia, Takayasu arteritis (TAK) is well-known. However, this dynamic is changing with the advent and increasing use of computed tomography angiography (CTA; Numano et al., 2000; Ogunbiyi and Falase, 1989; Hodes, 1990). Diagnosing TAK requires a high index of suspicion as the clinical presentation is protean and may be non-specific (Lamessa et al., 2024). Notably, the neurological manifestations of TAK are closely related to the specific vascular pathology and the arterial territory involved (Mirouse et al., 2022; Duarte et al., 2016). While acute ischemic stroke (AIS) is commonly due to stenosis/thrombosis of the middle and anterior cerebral arteries (branches of the carotid artery), anterior ischemic optic neuropathy (AION) results from inflammation and subsequent thrombosis of the short posterior ciliary arteries (SPCA), which originate from the ophthalmic artery (branches of the carotid artery; Patel and Miller, 2024).

Since the early reports by Takayasu, there have been several other descriptions of TAK in non-Asians and male individuals (Hodes, 1990; Patil and Rajoor, 2013), with a diverse range of clinical manifestations. In a recent retrospective multicenter study involving 320 patients with TAK, 41 (13%) had a stroke localized to the carotid artery territory in 87% of cases (Mirouse et al., 2022). Stroke symptoms were commonly hemiplegia, while visual loss, facial paralysis, and aphasia occurred infrequently. A recent meta-analysis of 3,262 patients with TAK documented that, in addition to stroke and myocardial infarction, other ischemic complications were inconsistently reported (Kim and Barra, 2018). A case series of four cases similarly documented lower limb claudication, treatment-resistant hypertension, subclavian artery stenosis, and stroke (ischemic and subarachnoid hemorrhage), posing varying degrees of diagnostic uncertainties (Box et al., 2021). AIS and AION, which can sometimes be the first manifestation of TAK, are rarely described (Mirouse et al., 2022; Kim and Barra, 2018). While there are isolated cases of AIS and AION (Mirouse et al., 2022; Schmidt et al., 1997; Lewis et al., 1993), no case report of concomitant AIS and AION as an initial manifestation of TAK exists. We describe a rare case of TAK causing AIS and AION in a young Nigerian woman. We also highlight the challenging diagnostic trajectory.

## Case presentation

A 33-year-old female patient was admitted (February 2020) to the emergency department of our hospital due to a 6-h medical history of abrupt onset weakness in the right extremities and loss of vision in her left eye following a syncopal episode. There was associated slurred speech and facial asymmetry. There was no preceding or accompanying headache, vertigo, gait imbalance, seizures, chest pain, or palpitations. She had no fever, facial (malar) rash, hair loss, or oral ulcerations. She had no history suggestive of recurrent first-trimester pregnancy losses, livedo reticularis, finger or toe discoloration, or intermittent claudication. There was no history of use of oral contraceptives or psychoactive substances. She had noted a preceding history of blurred vision before this admission with ringing in the ears. A Dix–Hallpike maneuver was negative, and the remainder of the otorhinolaryngology review was largely unremarkable. Approximately 12 months earlier, she had been seen on account of dizziness and syncopal attacks, with findings of low blood pressure that required repeated hospital admissions. Serial electrocardiogram, echocardiography, and random blood sugar tests were unremarkable. She was managed as a case of hypovolemic shock and received 4 liters of intravenous fluids without improvement. Her symptoms remained under control with postural adaptations, dietary modifications (fluids and small meals), and physical rehabilitation. There was associated generalized malaise, weight loss, and joint pain involving the right knee, neck, and lower back. Precisely 18 months before the current admission, during the first trimester of pregnancy, she noted numbness in both fingers and feet with pain, and a finding of low-volume radial pulse was documented. She was referred to the cardiology clinic with a diagnosis of possible congenital absence of the left subclavian artery. Vascular imaging was requested. She had a cesarean section a few months later and was lost to follow-up following childbirth. She had an appendectomy done in the past. She was not previously diagnosed with hypertension, type 2 diabetes mellitus, or stroke and did not take alcohol or smoke. There was no known family history of a similar illness.

At admission, the vital signs were unrecordable blood pressure (BP; left arm) and 80/50 mmHg (right arm), with a pulse of 82 per minute. The BP in the lower limbs was left (130/68 mmHg) and right (130/78 mmHg), with an ankle-brachial pressure index of 1.86 and 1.62, respectively. The general physical examination showed a conscious but anxious young lady who was not pale, not febrile, not cyanosed, well-hydrated, and with no significant peripheral lymphadenopathy or pedal swelling. The neurological examination noted a conscious lady with expressive aphasia. The right pupil was 3.5 mm, round, and reactive, while the left pupil dilated on exposure to bright light. There was no light perception in the left eye with Marcus Gunn's pupil (relative afferent pupillary defect). Fundoscopy showed a pale left optic disc, cup–disc ratio of 0.4, attenuated vessels, and normal macular (in keeping with acute left AION). There was no ophthalmoplegia. She had right supranuclear facial nerve palsy with right flaccid hemiparesis. The power in the left extremities was 5/5. The National Institutes of Health Stroke Scale score was 14. The cardiovascular examination showed a pulse (right radial) of 82 per minute, which was small in volume but regular. She had absent left radial and brachial pulsations, reduced right radial and brachial pulsations, and reduced carotid pulsations bilaterally, worse on the left. The lower limb pulses were normal. No bruit was detected on peripheral examination. Evaluation with ankle-brachial pressure index showed turbulent and biphasic flow in multiple arterial territories of the upper limbs. The BP was unrecordable in the left arm, while it was 80/50 mmHg in the right arm. The precordial examination findings as well as the heart sounds were normal. The abdominal examination as well as the chest findings were otherwise not remarkable. The initial suspicion was hypovolemic shock with likely watershed infarct.

### Investigations

**Figure 1:**
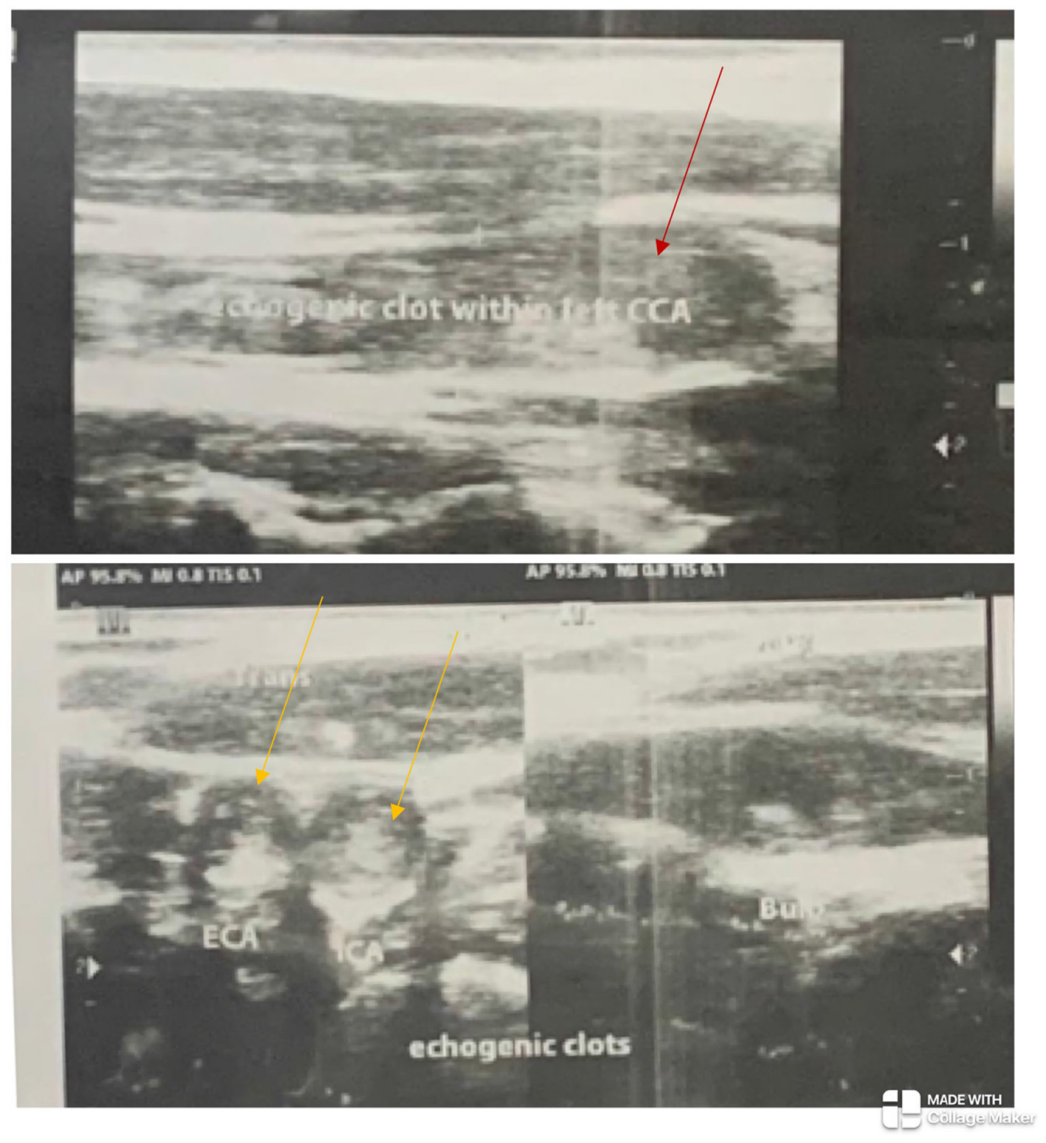
The left common carotid artery (red arrow) and the left internal and external carotid arteries (yellow arrows) show echogenic clots completely occluding their lumina with absent flow on Doppler ultrasonography.

**Figure 2:**
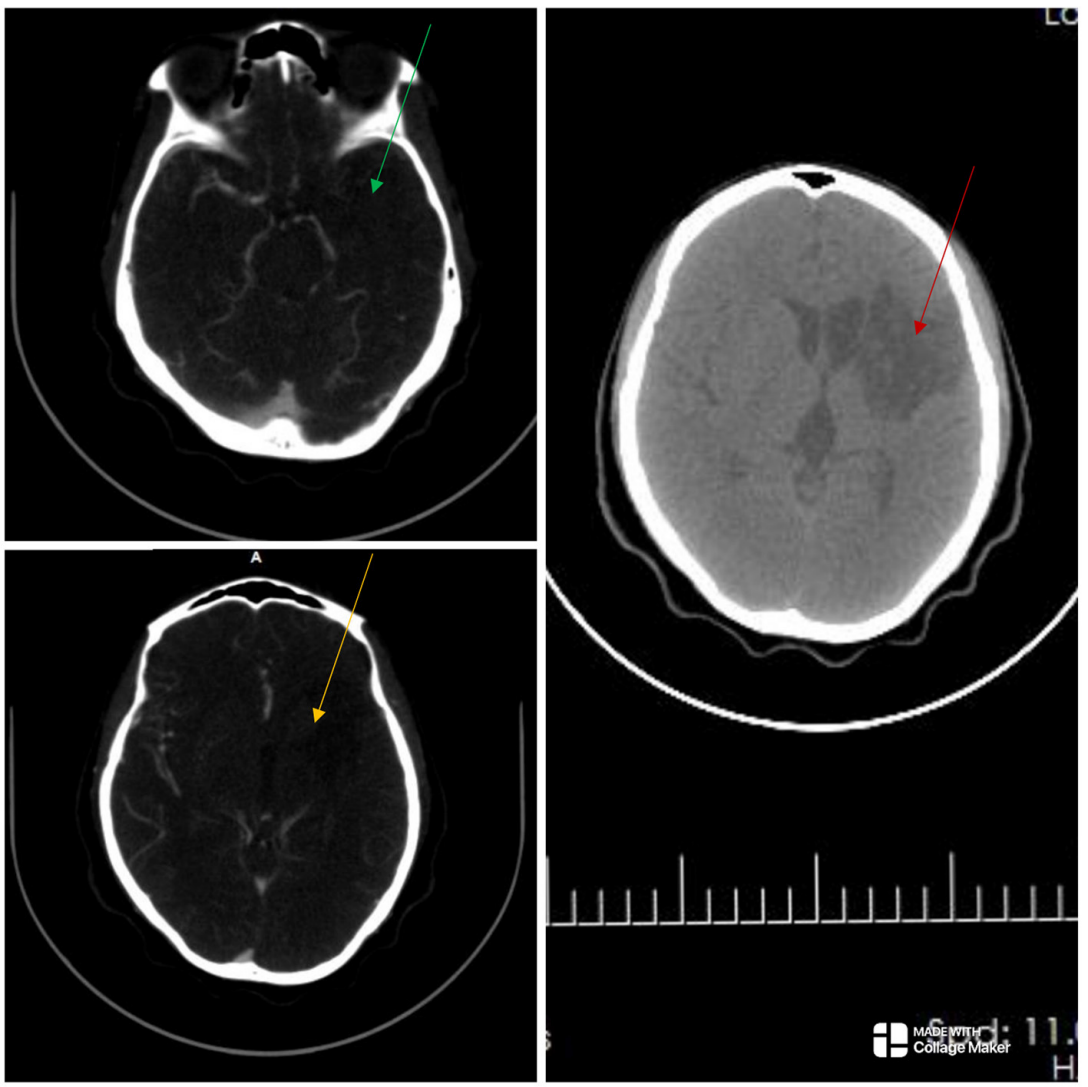
The cranial computed tomography (CT) scan **(right)** shows an ill-defined oval-shaped (red arrow), hypodense area (HU = 14–36) involving the left basal ganglia and frontal lobe in keeping with an acute infarct. The CT angiography **(left)** showed absent opacification at the origin of the right common carotid artery and a string-like lumen from C7/T1 to C4/5 (not shown). There was opacification at the origin of the left internal carotid artery (ICA) but the artery abruptly terminated just beyond that point. There was no opacification in the cervical or intracranial segments of the left ICA (green and yellow arrows), as well as in the left subclavian artery. There were short segment stenoses in the pulmonary artery branches bilaterally (not shown). Overall features showed diffuse vascular smooth muscle wall disease affecting both intracranial and extracranial vessels, in keeping with inflammatory vascular disease.

The electrocardiogram and echocardiogram were normal. Other laboratory findings are shown in Table 1. The carotid Doppler Ultrasonography (USS) showed complete occlusion of the left common carotid artery and left internal and external carotid arteries with absent flow on Doppler interrogation (Figure 1). The cranial CT scan (Figure 2) showed an ill-defined, oval-shaped hypodense area (HU = 14–36) involving the left basal ganglia and frontal lobe in keeping with an acute infarct. The brain CTA showed diffuse enhancing aortic arch wall thickening and multiple aortic arch branch obstructive disease (Figure 2). There were multiple smooth-walled, long-segment luminal stenoses but no aneurysms. Overall features showed diffuse vascular smooth muscle wall disease of both intracranial and extracranial vessels, in keeping with inflammatory vascular disease.

**Table 1:** Investigations done.

**Reference range**	**Ref. range**	**Feb. 2020**	**5 months later**
Hemoglobin (g/dl)	12–16	10.3	11.5
Hematocrit %	36.0–49.0	33	34.7
White cell count/mm^3^	4,000–11,000	8,100	13,610
Neutrophils (%)	37–72	51.8	66.5
Lymphocytes (%)	20–50	33	26.4
Monocytes (%)	2–10	7.5	6.6
Eosinophils (%)	0–6	6.7	0.2
Basophils (%)	0–1	1.0	0.4
Platelet count/mm^3^	150,000–400,000	305,000	284,000
ESR mm/first hour	1–20	81	50
RBG (mg/dl)	80–140		71
Sodium (mmol/L)	130–145	139	
Potassium (mmol/L	3–5	3.0	
Chloride (mmol/L)	95–110	106	
HCO_3_ (mmol/l)	20–30	20	
Urea (mg/dl)	15–45	22	
Creatinine (mg/dl)	0.5–1.5	0.7	
Total cholesterol (mg/dl)	<200		169
HDL (mg/dl)	>40		33
LDL (mg/dl)	<130		108
Triglyceride (mg/dl	<160		141
PT (s)	14–16		11.4
PT (control)	–		12.5
APTT (s)	20.7–36.5		35
INR	0.9–1.1		0.91

ESR, erythrocyte sedimentation rate; RBG, random blood glucose; HDL, high-density lipoprotein; LDL, low-density lipoprotein; PT, prothrombin time; APTT, activated partial thromboplastin time; INR, international normalized ratio.

### Diagnosis

The final diagnosis was TAK with complicating left middle cerebral artery (MCA) territory infarct and AION. Based on the 2022 American College of Rheumatology (ACR)/European League Against Rheumatism (EULAR) Classification criteria, our patient had a score of 9 (Grayson et al., 2022; Supplementary material 1). She also met the criteria using the modified Ishikawa score (Sharma et al., 1996; Ishikawa, 1988; Supplementary material 2), with a Numano type I angiographic extent of disease (Hata et al., 1996; Joseph et al., 2022). The Indian Takayasu clinical activity score (ITAS 2010) was 7 (suggestive of active disease; Misra et al., 2013).

### Care, outcome, and follow-up

She was managed by a multidisciplinary team of neurologists, rheumatologists, cardiologists, internists, and cardiothoracic surgeons. She was started on oral prednisone 1 mg/kg/day, tapered to 10 mg/day within 3 months, cilostazol 100 mg daily, aspirin 75 mg, clopidogrel 75 mg, atorvastatin, and dipyridamole 75 mg daily. She also received tabs of rabeprazole 40 mg twice daily for prophylaxis against gastric ulcers. At a follow-up review, the clopidogrel, atorvastatin, cilostazol, and dipyridamole were discontinued. On the subsequent visits, her symptoms had improved, and she was functioning well with a modified Rankin score (mRS) of 2. Her left vision improved to counting fingers. However, she noted hemoptysis months later, with radiologic findings of ground-glass opacification in both lung fields. No lung masses or cavitations were seen. Sputum testing for mycobacterium tuberculosis, microscopy/culture studies, and a COVID-19 screening were negative. She was managed for pulmonary vasculitic infarcts, and azathioprine 50 mg daily was added. Symptoms improved with the use of immunosuppressants and supportive care. Due to severe financial constraints, neither biologic disease-modifying antirheumatic drugs (DMARDs) nor revascularization therapy for carotid artery occlusion were commenced. She is currently being followed up in neurology, cardiothoracic, cardiology, rheumatology, and pulmonology clinics with an ITAS 2010 score of 1 (inactive TAK).

## Discussion

In this report, we described a rare case of a 33-year-old Nigerian female with indolent onset constitutional symptoms first noted 18 months before the first clinical suspicion of TAK. We highlighted the challenging diagnostic trajectory and the unusual multi-vessel ischemic complications, namely, AIS and AION. TAK is a rare large-vessel vasculitis of yet unclear etiology that leads to arterial wall thickening, stenosis, occlusion, and microaneurysm, which may be complicated by myriad cerebrovascular events (Mirouse et al., 2022; Kim and Barra, 2018; Numano et al., 2000). The prevalence of ischemic events has been inconsistently documented in the literature with varying information on the stroke subtype, characteristics of the affected individuals, and presence of stroke-associated morbidity and mortality (Duarte et al., 2016). Stenosis or occlusion of the branches of the internal carotid arteries, namely, the anterior artery and MCA, as well as the ophthalmic arteries, may lead to anterior circulation ischemic stroke and AION, respectively. The stroke mechanism may be thrombotic, embolic, or both (Field et al., 2017). Worthy of note is that our patient's symptoms were non-specific, ranging from generalized malaise, numbness, joint pain, fever, weight loss, and dizziness. Notably, she had multiple clinical encounters with the diagnosis and management of hypovolemic shock due to pulselessness in the extremities and unrecordable blood pressure (pseudo-hypotension; Hafner et al., 2012). Her dizziness and syncopal events were likely due to the underlying large vessel occlusion and hypotensive episodes. The thought of TAK did not come as a possible etiologic consideration for her myriad symptoms until she developed a concomitant stroke and AION. She had CTA done and met diagnostic criteria using both the ACR/EULAR and the modified Ishikawa criteria. Following diagnosis, she was commenced on secondary stroke preventive care as well as steroid therapy, which was subsequently transitioned to steroid-sparing agents. Reduced disease activity was evidenced by the lower ITAS 2010 score, good functional outcome, and improved vision.

In TAK, the non-specific clinical presentations and laboratory test results frequently contribute to late diagnosis and delayed treatment. In a retrospective 46-year multicenter study (the French Takayasu network), the median time from symptom onset to TAK diagnosis in that cohort was 0.8 (0–3.9) years (Mirouse et al., 2022). In that cohort, the time from first symptoms to TAK diagnosis >1 year was independently associated with cerebrovascular ischemic events (Mirouse et al., 2022). Similarly, a recent case series described unusual presentations of TAK with stroke, highlighting varying degrees of diagnostic delays with suggestions for a high index of suspicion (Box et al., 2021). None of these cases reported concomitant vision loss and stroke. A systematic review and meta-analysis showed that the overall pooled prevalence of stroke/transient ischemic attack among TAK patients was 15.8% (Duarte et al., 2016), similar to findings from a retrospective multicenter study (Mirouse et al., 2022).

Treating TAK entails pharmacological therapy or carefully selected vascular intervention (Field et al., 2017; Roy and Singhal, 2022). Due to a lack of funds, revascularization therapy for carotid artery occlusion was not available to our patient. Similarly, the often poor arterial access linked to multiple occlusive lesions and vessel friability in the setting of severe inflammation meant that aggressive medical management is more often embraced (Field et al., 2017; Ghoshal et al., 2016; Humayun et al., 2014). The goal of medical therapy is to control disease progression and prevent recurrent ischemic episodes (Gao et al., 2020). Glucocorticoids are effective agents for patients with active TAK. Our patient received prednisolone during the active phase of her illness and was later transitioned to azathioprine. Current guidelines on anticoagulation/antiplatelet therapy in TAK remain unclear, and practical results are conflicting (Field et al., 2017; Roy and Singhal, 2022; Gao et al., 2020). In a recent case report of TAK, aspirin was concomitantly used with warfarin effectively (Field et al., 2017), while some other authors advocate using dual-antiplatelet therapy. We favored an initial use of dual-antiplatelet therapy in this case with aggressive medical care (statins and cilostazol) given the extensive multi-vessel involvement and need to prevent recurrent ischemic events (Derdeyn et al., 2014; Kleindorfer et al., 2021). However, given the unimpressive cardiovascular workup for traditional stroke risk factors, the statins, clopidogrel, cilostazol, and dipyridamole were discontinued. The efficacy of conservative therapy in managing cardiovascular complications of TAK is not fully established and is a subject of ongoing research (Samaan et al., 2024; Bhandari et al., 2023; Putaala et al., 2017). Because thrombus formation can be provoked by the inflammatory milieu in TAK, controlling the ongoing inflammation is paramount and requires an individualized approach (Gao et al., 2020; Bhandari et al., 2023).

Most reports of TAK from Africa have emanated from northern and southern Africa, likely due to the relative availability of advanced radiologic/contrast imaging studies (Genga et al., 2018). However, the dynamics are changing with the increasing availability of radio-diagnostic modalities. Fairly recently, a Nigerian author reported that TAK often presents with atypical features of ulnar artery occlusion (Odunlami et al., 2020). Another case has been reported in a young Nigerian boy with generalized body weakness, fever, and joint pain. None of these case reports noted concomitant features of stroke and visual impairment attributable to the central nervous system (Oguntona, 2010). Patil and Rajoor (2013) reported an asymptomatic 21-year-old male, with a finding of discrepant BP and pulse in the arms. There is a recent report of a 25-year-old Ethiopian woman who had a retrospective diagnosis of TAK 2 years after having had a stroke (Lamessa et al., 2024). The differential diagnostic considerations in our case included systemic lupus erythematosus (SLE), antiphospholipid syndrome, and chronic granulomatous infection with small-vessel vasculitis. Negative clinical findings of fever, malar rash, hair loss, mucosal ulcerations, recurrent first-trimester pregnancy losses, livedo reticularis, and finger or toe discoloration made these possibilities less likely. These were further corroborated by laboratory findings of normal prothrombin Time/activated partial thromboplastin time (PT/aPTT) and a negative sputum screen for infection. Besides, the CTA findings seen in our case are not characteristic of small vessel vasculitis, SLE, or secondary vasculitic syndromes from chronic infections.

TAK's etiology is assumed to be from a cell-mediated inflammatory process within the vasculature, which can result in occlusion, aneurysmal dilatation, and constriction in afflicted segments because of mononuclear and granulomatous infiltrates. Our patient had involvement of multiple arterial territories, manifesting clinically with acute stroke (left MCA territory) and AION (left ophthalmic artery). She also developed hemoptysis, with radiologic findings of ground glass opacification in both lung fields—a rare manifestation that could be from pulmonary arteritis, hypertension, and lung infarcts (Yang et al., 2019). When evaluating the hemoptysis, our considerations were infections (TB, bacterial pneumonia, and COVID-19 pneumonia), coagulation/bleeding disorders, or inflammation (from the underlying pulmonary arteritis). The infection and coagulation screen [prothrombin time/international normalized ratio (PT/INR) and platelet count] were otherwise unremarkable. Symptoms improved with the use of immunosuppressants and supportive care.

The lessons from our case are protean and profound. Detailed peripheral arterial examination remains a cardinal part of the cardiovascular system examination and should be emphasized at all levels. In cases of low blood pressure and suspected peripheral vascular disease, blood pressure recordings should be taken on both arms and compared (Hafner et al., 2012). The diagnosis of TAK should be made at an early stage before ischemic manifestations become obvious (Mirouse et al., 2022; Numano and Kobayashi, 1999). Because large-artery biopsies cannot easily be done (as in our case), the gold standard is angiography (Grayson et al., 2022; Hata et al., 1996). Angiography is non-invasive and readily acquired. Doppler ultrasonography and non-invasive MRA, however, can also produce results that are just as good. Multidetector CTA is an emerging diagnostic tool and may facilitate detecting vasculitis during the early phase of TAK. The prognosis of TAK varies widely and depends on the duration of illness before diagnosis, specific patient characteristics, the presence of vascular events or complications, and the severity of accompanying neurologic deficits (Mirouse et al., 2022; Duarte et al., 2016; Samaan et al., 2024). Our patient had a relatively good prognosis with mild neurologic impairment, limiting her ability to carry out all previous activities (mRS score of 2). However, her visual impairment improved only minimally to counting fingers, thus further worsening the morbidity. Early recognition and diagnosis are crucial to reducing attendant morbidity and mortality in TAK.

## Limitation and strength

The major limitation of our case is our inability to demonstrate the potential benefit of treatment options such as biologic DMARDs and revascularization therapy in our patient as these treatment modalities were unavailable due to financial constraints. Similarly, because large-artery biopsies are rarely advocated and highly invasive with attendant risks, the diagnosis of TAK was established using CTA (Grayson et al., 2022; Hata et al., 1996). Despite the forgoing constraints, our case sheds light on the need for early identification and recognition of the otherwise subtle symptoms and signs of TAK among ethnic groups, hitherto known to be unlikely to have TAK, while advocating for a high index of suspicion among that population. Given that there are likely a few more undiagnosed cases of TAK in the subregion, our report will help raise awareness and educate clinicians in these settings to identify cases of TAK before adverse cardiovascular complications set in. Notably, our case also uniquely describes a rare concomitant multi-vessel ischemic complication of TAK that is hitherto rarely documented.

## Conclusion

This case illustrates the complex diagnostic path of TAK in a Nigerian female with unique multi-vessel involvement. We documented concurrent AIS and AION, showcasing a rare instance of simultaneous occlusion of the left MCA and the ophthalmic artery, both branches of the internal carotid artery. The non-specific clinical presentation and low index of suspicion often result in late diagnosis and delayed treatment. Therefore, clinicians should be vigilant about the varied presentations of TAK to mitigate adverse cardiovascular complications.

## Data Availability

The original contributions presented in the study are included in the article/Supplementary material, further inquiries can be directed to the corresponding author.
