# Supplementary material for: Case report: A rare case of catastrophic Takayasu arteritis: acute ischemic stroke and anterior ischemic optic neuropathy
Source: Front Stroke. 2024 Nov 28;3:1481940. doi: 10.3389/fstro.2024.1481940 (PMC12802768; doi:10.3389/fstro.2024.1481940)
Supplement: Supplementary file 1 [file Table_1.docx]

SUPPLEMENTAL FILES

Supplemental file 1: 2022 AMERICAN COLLEGE OF RHEUMATOLOGY/EULAR^14^

| 2022 AMERICAN COLLEGE OF RHEUMATOLOGY/EULAR |  |
| --- | --- |
| ABSOLUTE REQUIREMENTS |  |
| Age ≤60 YEARS ** |  |
| Evidence of vasculitis on imaging ** |  |
| ADDITIONAL CLINICAL CRITERIA |  |
| Female sex** | +1** |
| Angina or ischemic cardiac pain | +2 |
| Arm or leg claudication | +2 |
| Vascular bruit | +2 |
| Reduced pulse in upper extremity** | +2** |
| Carotid artery abnormality** | +2** |
| Systolic blood pressure difference in arms >= 20mmHg** | +1** |
| ADDITIONAL IMAGING CRITERIA |  |
| Number of affected arterial territories (select one) |  |
| One arterial territory | +1 |
| Two arterial territories | +2 |
| Three or more arterial territories** | +3** |
| Symmetric involvement of paired arteries | +1 |
| Abdominal aorta involvement with renal or mesenteric involvement | +3 |

**met the criteria of TA diagnosis in our case.
