# Supplementary material for: Case report: A rare case of catastrophic Takayasu arteritis: acute ischemic stroke and anterior ischemic optic neuropathy
Source: Front Stroke. 2024 Nov 28;3:1481940. doi: 10.3389/fstro.2024.1481940 (PMC12802768; doi:10.3389/fstro.2024.1481940)
Supplement: Supplementary file 2 [file Table_2.docx]

SUPPLEMENTAL FILE 2

| Modified Ishikawa criteria for diagnosis of Takayasu arteritis. |
| --- |
| Major criteria  1. Left mid-subclavian artery lesion**  2. Right mid-subclavian artery lesion  3. Characteristic signs and symptoms of at least 1-month duration**  Minor criteria  1. High ESR: Unexplained ESR > 20 mm/h**  2. Carotid artery tenderness  3. Hypertension; Persistent BP >140/90 mm Hg brachial/>160/90 popliteal  4. Aortic regurgitation or annulo-aortic ectasia  5. Pulmonary artery lesion**  6. Left mid-common carotid lesion**  7. Brachiocephalic trunk lesion  8. Descending thoracic aorta lesion  9. Abdominal aorta lesion  10. Coronary artery lesion |

Adapted from Sharma et al.^15^, modified from Ishikawa^16^.

BP: blood pressure; ESR: erythrocyte sedimentation rate.

The presence of 2 major or 1 major and 2 minor or 4 minor criteria suggests a high probability of Takayasu arteritis.

**met the criteria of TA diagnosis in our case.
